# Attention as foraging for information and value

**DOI:** 10.3389/fnhum.2013.00711

**Published:** 2013-11-05

**Authors:** Sanjay G. Manohar, Masud Husain

**Affiliations:** ^1^Department of Experimental Psychology, University of OxfordOxford, UK; ^2^Nuffield Department of Clinical Neurosciences, John Radcliffe HospitalOxford, UK

**Keywords:** attention, saccades, foraging, uncertainty, information, expected value, risk, bayesian updating

## Abstract

What is the purpose of attention? One avenue of research has led to the proposal that attention might be crucial for gathering information about the environment, while other lines of study have demonstrated how attention may play a role in guiding behavior to rewarded options. Many experiments that study attention require participants to make a decision based on information acquired discretely at one point in time. In real-world situations, however, we are usually not presented with information about which option to select in such a manner. Rather we must initially search for information, weighing up reward values of options before we commit to a decision. Here, we propose that attention plays a role in both foraging for information *and* foraging for value. When foraging for information, attention is guided toward the unknown. When foraging for reward, attention is guided toward high reward values, allowing decision-making to proceed by accept-or-reject decisions on the currently attended option. According to this account, attention can be regarded as a low-cost alternative to moving around and physically interacting with the environment—“*tele*foraging”—before a decision is made to interact physically with the world. To track the timecourse of attention, we asked participants to seek out and acquire information about two gambles by directing their gaze, before choosing one of them. Participants often made multiple refixations on items before making a decision. Their eye movements revealed that early in the trial, attention was guided toward information, i.e., toward locations that reduced uncertainty about value. In contrast, late in the trial, attention was guided by expected value of the options. At the end of the decision period, participants were generally attending to the item they eventually chose. We suggest that attentional foraging shifts from an uncertainty-driven to a reward-driven mode during the evolution of a decision, permitting decisions to be made by an engage-or-search strategy.

## Introduction

Recent studies have suggested that visual attention might play a role both in acquiring information and searching for reward. Several groups have demonstrated that reward can guide attention (Ding and Hikosaka, 2007; Hickey et al., 2010; Anderson et al., 2011; Schütz et al., 2012; Camara et al., 2013). Others have argued that attention needs to be drawn to stimuli that have a high uncertainty to facilitate acquisition of information (Yu and Dayan, 2005; Hogarth et al., 2008; Gottlieb and Balan, 2010). Acquiring information by directing attention is an active, dynamic process (Ballard et al., 1995; Shinoda et al., 2001), where information is the reduction of uncertainty in our estimate of world states or future outcomes (Feldman and Friston, 2010).

Which of these two drives, reward or uncertainty, controls the shifts of attention before a decision? Information integration for decisions has been the objective of a wealth of neuroscientific studies (e.g., Platt and Glimcher, 1999; Shadlen and Newsome, 2001; Smith and Ratcliff, 2009; Basten et al., 2010; Hare et al., 2011), but surprisingly little research has focused on the dynamic control of attention while searching *for information* (Reutskaja et al., 2011; Gottlieb, 2012). In most experimental situations, observers simply choose between two options at a discrete point in time, but are not allowed to sample the environment and integrate different types of information as they might naturally, over time.

Behavioral ecology, by contrast has concerned itself with how animals sample the environment (forage) before coming to a decision (Krebs et al., 1978; Stephens, 1987; Stephens and Krebs, 1987). Here we present a new experimental paradigm that allows us to compare how attention is directed to reward, risk, and uncertainty about reward. We then discuss a framework in which attentional guidance shifts during choice, from information-driven, to reward value driven.

Attention influences decision processes both by selecting which information is accumulated in decision variables (Einhorn and Hogarth, 1981; Roe et al., 2001; Krajbich et al., 2010), but also by biasing choice toward the attended option (Shimojo et al., 2003; Brandstätter, 2011). But what guides attention itself? Unless carefully guided, attention would be maladaptive, biasing information and choice. When attention biases choice, attending to the higher expected value (*EV*) might be beneficial; whereas when attention determines which information is gathered, then attending to uncertainty might be beneficial (Itti and Baldi, 2009). Although information-seeking may ultimately help to obtain reward, we distinguish it from “value-driven” guidance in which attentional is directly attracted toward reward.

Information could drive attention in two possible ways. A *perceptual* model of attention predicts that we focus on items that have greater uncertainty in their *identity* (Feldman and Friston, 2010). However, an *action*-driven model of attention would require that we focus on items that have greater uncertainty in their *value*. In other words, attention's primary role might be to provide decision making systems with information about the *EV* of the options being considered (Gottlieb and Balan, 2010), and thereby reduce risk.

Neither of these information-driven models explains the finding that, in choice, we generally choose the item we were last attending to (Krajbich et al., 2010), at least when the attended item is more valuable than the alternatives. We suggest that this tendency, although intuitive, requires explanation, and reveals key features of the tight link between attention and choice. A parsimonious explanation of this phenomenon is to regard attention as a form of *foraging*.

Rather than simply deciding which item is better, we argue that decisions are made by an “engage or search” strategy. Unlike classical decision-making models, this captures the intuition that we rarely choose something we are not attending to (Reutskaja et al., 2011). Attentional shifts, then, can be viewed as a low-cost alternative to physically moving around an environment before engaging with the world. In other words, attention might be a mechanism of “*tele*foraging”: gathering and evaluating information at a distance before physically engaging with the environment.

In such a model, when we are free to search for information, attention would be considered to be driven *both* by uncertainty and *EV*, to jointly achieve the goals of information acquisition, and option selection. Option selection is then framed as either accepting the currently attended option (“engage”) or moving to the other location (“search”). From this perspective, any progressive reduction of uncertainty by guiding attention can be viewed as “foraging for information.”

**Figure 1:**
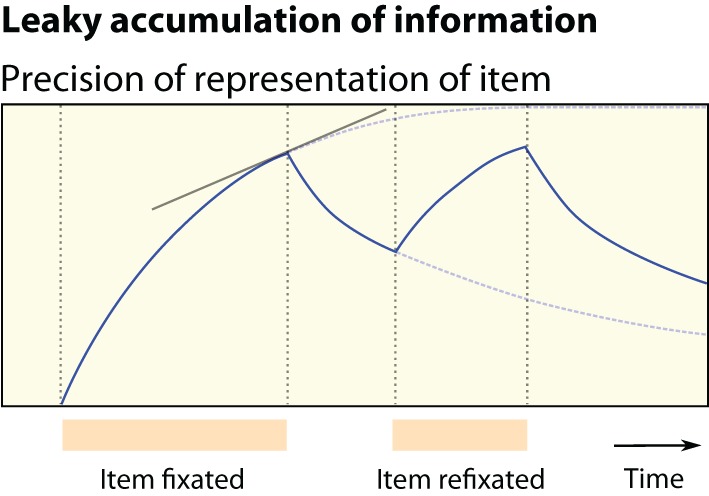
**Foraging for information**. We test the view that foraging for information involves the leaky accumulation of information about the fixated item. Information acquisition involves a time-dependent, location-specific *gain in precision*. Participants should leave a location when the information gain rate falls below a threshold, in parallel with classical foraging for reward (Stephens and Krebs, 1987). The location fixated next is determined by which location has the greatest estimated information gain rate. Meanwhile information about the original item *decays*. This predicts that participants refixate the first item seen, that dwell times shorten over the course of a trial, and that longer fixations result in fewer subsequent refixations of the same item.

Foraging for food involves deciding, after each movement, whether to engage a current option, or to move off and continue the search (e.g., Charnov, 1976; Kolling et al., 2012). Foraging for information, we propose, might involve deciding *at each fixation* whether information is sufficient to support choosing of the attended option, or not. Critically, over the course of each individual fixation, we might expect the amount of information being acquired to decrease (Figure 1). Thus, attention might shift to a new location when the information rate drops below a threshold, in parallel with animal models of foraging for reward (Waage, 1979; Stephens and Krebs, 1987).

Viewed as foraging, information acquisition would be expected to show a characteristic timecourse. Exploration during foraging is driven by our estimates of uncertainty in a variable environment (Behrens et al., 2007), so rather than simply attending to the highest expected value, a *systematic exploration* of the options would be envisaged to occur, perhaps described by an analog to the optimal departure rule developed for animal foraging (Pyke, 1978). Furthermore, according to this view, options might also be revisited, as needed, to acquire more information (Waage, 1979; Pyke, 1984; Gill, 1988).

But later during a decision process, the marginal information yield (reduction in uncertainty) of an attentional shift should become small (Figure 1) as less information is gained with each new fixation (Armel and Rangel, 2008). Therefore, according to this perspective, we would anticipate that attention becomes progressively more governed by expected value and guided toward the more valuable option. This schema allows a foraging-type *“accept or reject” decision* to be made at each fixation, culminating in the selection of an option.

An alternative way of putting this hypothesis is that under conditions of uncertainty, *information* carries salience, but as more information is acquired, *reward value* should become salient. The allocation of attention during a decision is initially uncertainty-driven, but as information is “consumed,” and *EV* estimates become more precise, *EV* itself guides attention, culminating in choice of the attended option. Such dynamic changes in attentional guidance could resolve a longstanding rift in the attention literature, between those that demonstrate attention to uncertainty, vs. those showing that reward guides attention.

**Figure 2:**
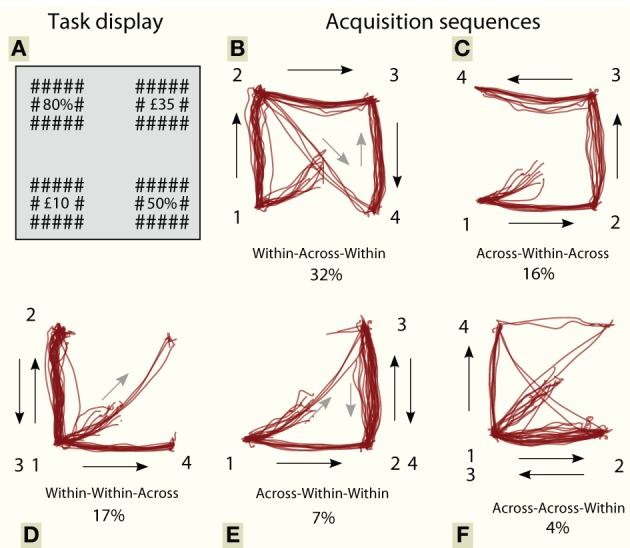
**(A)** Our task is a choice between two gambles, presented on the left and right hand sides of the screen. Participants freely viewed a display with four numbers, to acquire information about the two gambles. Without a time limit, they selected the preferred gamble by a keypress. Each gamble had a probability of winning vs. losing, denoted with a “%” suffix, and a monetary stake, denoted with a “£” prefix. After selection, a sound indicating win/lose was played over a loudspeaker, and the bank balance was displayed centrally. The numbers were small and were presented close to isoluminance, ensuring that fixation was necessary to identify numbers. **(B–F)** Example scan paths of the first four acquisitions from one participant, aligned so that the first saccade is to the lower left. Trajectories are classified according to the fixation pattern: each of the three saccades could either be within an option or across options. Numbers represent order of acquisition.

We designed a task specifically to examine the timecourse of attentional control before a decision is made. In our design, participants are allowed to *forage for information* from a limited set of risk and reward data for as long as they like before they make their decision. By tracking their eye movements we can obtain a measure of where, how and in what order attention is deployed over time prior to a decision. Participants viewed two gambles, on the left and right of the screen, each of which was characterized by a probability and a monetary stake, displayed numerically on a vertical axis (Figure 2A). They had to fixate these four numbers to acquire information about the two gambles, importantly *without* any time limit, before they chose one of the two gambles by a keypress.

**Figure 3:**
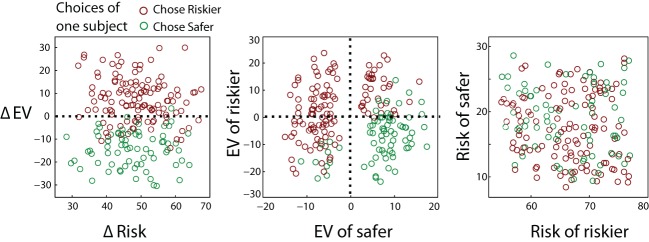
**Trials were chosen to give a spread of expected values (*EV*s) and a spread of risks**. One gamble always had a high risk, and the other a low risk. On some trials the choice was easy (small *EV* difference), on others it was hard (large *EV* difference). Colors demonstrate the choice on each trial for one representative participant, showing near-optimal choice.

After choosing, they either won or lost the stake of the chosen gamble, with the specified probability of winning. Thus, choosing a probability greater than 50% was likely to win the stake, whereas below 50% was likely to lose money. A range of expected values and risks were chosen for each gamble. One gamble was always more risky than the other, but could have a higher or lower *EV* than the safer gamble (Figure 3). This allowed us to describe the trajectory of attention in terms of the relative “pull” of *EV* and uncertainty (composed of gamble risk and *EV* variance).

## Materials and methods

### Participants

In our task, participants had to make a choice between two gambles, but were given unlimited time to come to a decision. The gambles were presented on the left and right hand sides of the screen and participants freely viewed a display with four numbers, two on either side of the screen, to acquire information about the two gambles. Each gamble was given a *probability* of winning vs. losing (denoted with a “%” suffix) and a monetary *stake* (denoted with a “£” prefix). Both the probability and stake associated with a gamble were presented separately, one above the other (location randomized). Participants selected their preferred gamble by a keypress. After selection, a sound indicating win/lose was played over a loudspeaker, and the “bank balance” was displayed centrally, which was either incremented or decremented by the chosen stake. We recruited 17 participants from an advert, mean age 41. Research was conducted with informed consent, and was approved by the Imperial College Research Ethics Committee.

### Stimuli

Stimuli were displayed in Matlab and PsychToolbox on a CRT at 1024 × 768 pixels, 100 Hz. Participants had to fixate a central cross before the start of each trial. Numbers were displayed in the four quadrants of the display, at an eccentricity of 10°, with size 0.5°. Probabilities were indicated with a “%” suffix, and monetary stakes were indicated with a “£” prefix (Figure 2A). In order to ensure that identifying a number required fixating it, all numbers were two digits long, were masked by “#” symbols on all four sides, and were close to isoluminance with the background.

Fifty percent of the trials were *“colour-coded,”* such that probabilities were in one color, and stakes in another, with the code being consistent for each participant (counterbalanced). Participants were informed of these color contingencies before the experiment. Thus, in the color-coded trials, they could know whether each location contained a probability or a stake, in advance of fixating it. This allowed us to examine whether participants could utilize such prior knowledge to strategically fixate items of the same “dimension” (stake or probability) when looking between options.

During the decision period, an Eyelink 1000 Hz infrared eye tracker allowed us to follow the sequence in which numbers were fixated over the decision period. Participants then made a choice by pressing a left or right key with the index or middle finger of their right hand. When the choice was made, an auditory tone of high or low pitch indicated whether participants had won or lost, and after 1500 ms the running total (bank balance) amount won was displayed in the center of the screen for 1 s. Participants had to fixate a central cross for 500 ms prior to the start of the next trial. Participants completed two 64-trial blocks over 30 min and were paid based on their winnings.

We analyzed fixations in the period from display onset to choice keypress. We removed blinks and discarded fixations shorter than 50 ms and fixations off the display items. The item fixated at any time was determined with an 8° radius. Blinks accounted on average for 2.4% of decision time, and off-item fixations accounted for 3.8% of the decision time. Dwell times were calculated as the time between arriving at an item, and arriving at the next item.

### Gambles

The probability and stake for each gamble gives an expected value (*EV*) and a risk (*R*). Here, risk is defined as variance or uncertainty in the outcome:

(1) EV=S·(2P−1)

where *S* is stake and *P* is probability of winning. Note that the factor 2P − 1 incorporates the possibility of both winning and losing the stake. Probabilities under 50% yield a negative *EV*. From Equation (1), we can see that a gamble with a 50% *probability* of winning or losing has *EV* = 0. At the start of a trial, both P and S are uncertain, but after acquiring information, they will be more precisely known. Therefore, we can consider both S and P as random variables that must be estimated by the brain.

Of note, *knowing only the probability* gives information about the expectation of *EV*, whereas *knowing only the stake* does not: the expectation of *EV* remains zero. For example, knowing whether the stake is £10 or £90 makes no difference to participants' (mathematical) expectation of reward, because they could either win or lose it.

Next, we can calculate gamble risk, defined as variance of reward value:

(2) $$R=4S^{2}P(1-P)$$

According to this equation, a probability of 50% carries the highest risk because the outcome is most uncertain, and as probabilities get closer to 0 or 100%, the outcome is more predictable, so risk falls. Notably, the expectation of risk also changes when we learn a stake (unlike our expectation of *EV*)—i.e., after seeing a £90 stake, the risk estimate is high, since the outcome value is highly variable: +£90 or −£90.

On each trial, one of the two gambles had a high risk, and the other had a low risk (Figure 3). Values were chosen using four trial types, where the risky *EV*/safe *EV* were +8/+8, +8/−8, −8/+8 or −8/−8. Each of the four values (two probabilities and two stakes) was then randomized by adding a uniformly distributed integer from −10 to +10. This gave a set of trials which had a spectrum from similar *EV*s to different *EV*s, and high to low *EV*s. Similarly, risks ranged from high to low, with the difference in risks ranging from 20 to 70. The risky gamble's stake was between 57 and 77, and the safe stake was 10–30.

## Results

### Pre-choice behavior

**Figure 4:**
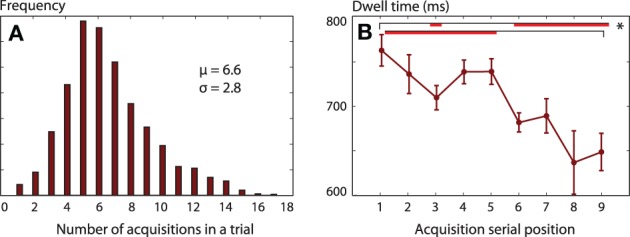
**(A)** Average histogram of the number of acquisitions (periods contiguously fixating one number) on each trial. Participants usually make four or more acquisitions, but sometimes require 14. **(B)** Dwell times decrease during the course of a trial. The final acquisition of each trial was excluded. Mixed-effects One-Way ANOVA showed a main effect of acquisition serial position in the trial, and the red bar shows pairs of significant differences (*p* < 0.05).

During the decision period, we traced the order of acquisition of information (one subject's first 4 fixations are shown in Figure 2B–F). “Acquisition” was defined as a period during which gaze remained on a single number (stake or probability), before moving to a different quadrant. Each acquisition lasted between 85 and 1800 ms, and could constitute several consecutive re-fixations around one particular item. Participants visited all four locations on 89% of trials. An optimal strategy might be to make only four acquisitions—provided that working memory can store four items, as some have argued to be the case (Cowan, 2010). However, we found that participants made on average 6.6 acquisitions before coming to a decision, and sometimes required up to 14 (Figure 4A).

In other words, they frequently refixated items prior to making a decision. One might predict that on this task, participants would visit all four locations before refixating any of them, consistent with “inhibition” of visited locations seen in visual search (Gilchrist and Harvey, 2000; Weger and Inhoff, 2006). However, our data showed, surprisingly, that on 49% of trials participants made refixations to a previously examined location *before* they had visited all four locations.

Mean dwell time on each acquisition was 762 ms and this decreased systematically over the course of a trial (Figure 4B). In this and subsequent analyses of fixation duration, we excluded the final acquisition during which the button-press choice was made, because the duration of this final fixation was presumably not determined by attentional search processes, but rather by action initiation. Dwell time on the first item was longer when a high *stake* was fixated, compared to a low stake [stake > median of £41, mean difference 33 ms, *t*_(16)_ = 2.18, *p* = 0.045]. The gamble's *probability* had no effect on dwell time (*p* = 0.38). Thus, at the start of a trial, gaze—and by inference, attention—appeared initially to be attracted by higher risk (since stake determines the variance in outcome) but not by higher *EV*.

### Choice behavior

**Figure 5:**
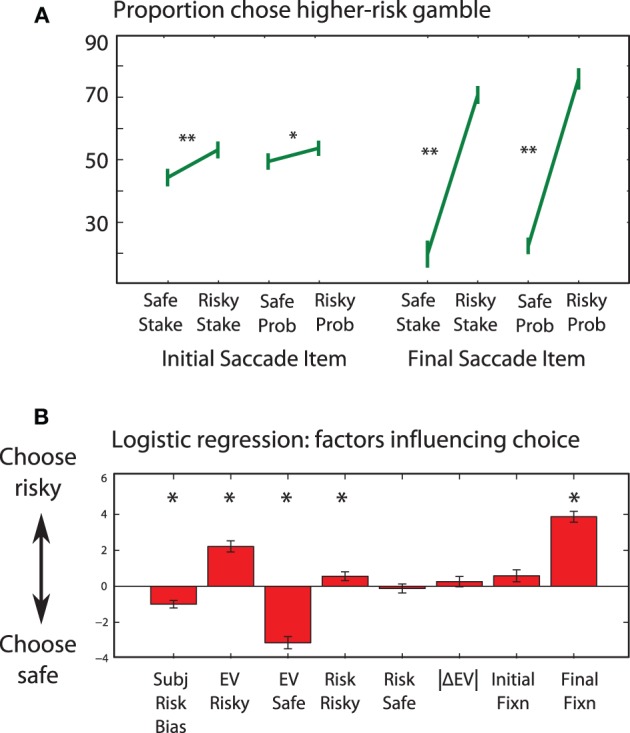
**(A)** Did attention correlate with choice? The first acquisition (left) predicts subsequent choice, despite being uncorrelated with any of the values seen. This demonstrates that participants are reliably biased by the first information they acquire. The final acquisition (right) strongly reflects the choice that is about to be made, with an accuracy of close to 80%: participants rarely choose an option they are not attending to. **(B)** Which factors influenced choice? An 8-factor model logistic regression model was fitted to each subject's choices, i.e., whether they chose the risky or safe option. We included included a bias term indicating individual risk preference, *EV* and risk of each option, and also eye movement factors from panel 5A—indicating whether the first and last fixations on each trial were to the risky option. The mean fitted normalized regression coefficients are shown. Error bars are s.e.m. across subjects. Asterisks indicate a regressor is significantly different from zero using *t*-test across subjects (*p* < 0.05). The initial fixation regressor was correlated with the final fixation regressor, and did not significantly contribute to choice on this analysis.

Participants chose the higher-*EV* gamble on 69% of trials overall. This occurred more often on “easy” trials—i.e., when the *EV*-difference between choices was large (absolute *EV* difference > median of £11: 77 vs. 61%, main effect of *EV* difference, *p* < 0.001). The higher *EV* was chosen less often when the risk difference was large (64 vs. 74%, main effect of risk difference, *p* = 0.03). Participants took less time to choose between the options when *EV*s were similar and large. There were strong biases for participants to choose the first option they fixated (*p* < 0.001) or the last item fixated (*p* < 0.001, Figure 5A), consistent with previous reports (Krajbich et al., 2010). This was despite the first saccade being directed essentially randomly (probability of 25% +/− 2% to each type of item, probabilty or stake, high or low value, *p* > 0.5), even when informative color coding (see Methods and below) was present. Logistic regression revealed that preference was governed primarily by *EV* difference but was also influenced by final fixations [both *t*_(16)_ > 7, *p* < 0.001, Figure 5B]. The preferred option consistently received more fixations and longer fixations, also consistent with previous findings (Glöckner and Herbold, 2011).

In our experimental design, 50% of the trials were “colour-coded,” such that probabilities were consistently in one color, and stakes in another. Thus, in the color-coded trials, participants could know whether each location contained a probability or a stake, in advance of fixating it.

If participants used this color information to guide attention, we might expect more horizontal saccades compared to diagonal saccades when corresponding dimensions (probability or stake) were aligned horizontally, and the converse when they are aligned diagonally. We found that although horizontal saccades were always more likely than diagonal saccades, there was no effect of display alignment (*t*-test of proportion of between-option saccades that were horizontal, *p* > 0.05), indicating that participants did not use color information in attentional guidance.

Choice reaction times were significantly faster when color-coding was present [4.32 vs. 4.69 s, *F*_(1, 16)_ = 8.88, *p* = 0.009], irrespective of whether the probabilities and stakes were horizontally or diagonally aligned. The advantage of color-coding was also evidenced by shorter durations of acquisitions (736 vs. 836 ms for the first acquisition).

### Information foraging

To analyse the data further we next developed a method to consider how information about *EV* is acquired over multiple fixations. A foraging account of attention postulates that the rate of acquiring new information decreases as participants gain greater knowledge about the fixated target (Figure 1).

(3) $$\begin{array}{l} \;k_{1}\frac{dI}{dt}=I_{\max}-I\\ \text{Rate of gain of information}\propto 1-\text{information already known} \end{array}$$

Once the information gain rate drops below the average information gain rate in the task, participants would be expected to direct attention to a new location, according to the marginal value theorem developed for foraging behavior (Charnov, 1976).

To explain refixations, we further assume that, after attention has left, the entropy of the posterior gradually rises, as information is lost. In other words, there would be a *natural decay*:

(4) $$\begin{array}{l} \;k_{2}\frac{dI}{dt}=-I\\ \text{Rate of loss of information }\propto \text{ amount of information currently known} \end{array}$$

With the assumption of decay, refixations can be explained by a rule that moves attention toward the unknown (toward high entropy). This information foraging account predicts that:

***(P1)*** Participants are more likely to revisit locations that were visited longer ago, because as the information decays, that location attracts more attention.
***(P2)*** Refixations are shorter than new fixations, because information is already (relatively) high at the start of fixation.
***(P3)*** Items that were fixated for longer periods are refixated fewer times, because participants have more information about those locations, and attention is drawn there less.

All three predictions turned out to be borne out by the results.

One might predict that on this task, participants would visit all four locations before refixating any of them, consistent with “inhibition” of visited locations seen in visual search (Gilchrist and Harvey, 2000; Weger and Inhoff, 2006). However, our data showed, surprisingly, that on 49% of trials participants made refixations to a previously examined location *before* they had visited all four locations.

#### Refixations go to locations fixated longer ago (P1)

At each fixation, we calculated the recency with which each display item was previously seen—i.e., how many items ago it was last fixated. On acquisitions that were refixations, the recency of the *fixated* item was 3.13 (*SD* 0.29). This compared with a recency of 2.72 (*SD* 0.13) for the other two items that were not selected by that eye movement [*F*_(1, 19)_ = 16.9, *p* < 0.001]. Thus, participants preferentially refixated items that had *not* been seen recently. The effect can be equally explained by foraging or inhibition of return.

#### Refixation durations compared to new fixations (P2)

**Figure 6:**
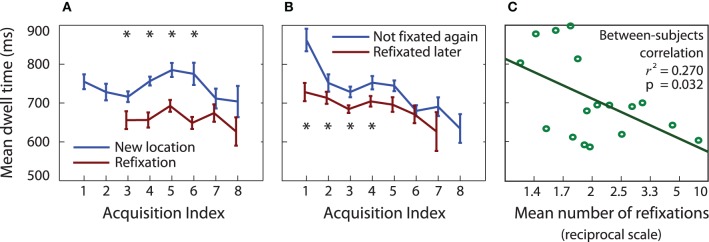
**(A)** Dwell times on previously unfixated items are longer than for previously fixated items (ANOVA main effect of previous refixation, *p* < 0.01; pairwise *t*-tests *p* < 0.01 at acquisitions 3–6). **(B)** Dwell times are longer when the item is never fixated again, compared to when it is fixated again later in the trial (ANOVA main effect of future refixation, *p* < 0.05; *t*-tests *p* < 0.05 at acquisitions 1–4). **(C)** Participants who made more refixations had shorter dwell times on average.

Refixations were shorter than acquisitions at unvisited locations even when they occurred at the same serial position in the trial [Figure 6A, *t*_(16)_ > 2.8, *p* < 0.01 at serial positions 3, 4, 5, and 6], just as might be predicted from a foraging perspective. This finding suggests that once viewed, an item cannot hold attention for as long.

#### Initial fixation time affects subsequent refixation duration (P3)

Initial dwell times were shorter at a location that was later refixated, compared to locations that were not refixated, even for acquisitions at the same serial position within a trial (Figure 6B, *p* < 0.01 for acquisitions at serial positions 1 and 2; *p* < 0.05 at 3 and 4). Thus, items that were briefly viewed were more likely to be refixated. This is in keeping with less information being accrued on shorter acquisitions (Figure 1). Participants who made shorter fixations on average also made more refixations (regression of mean dwell time over first four acquisitions against 1/(number of refixations), transformed to remove positive skew, *r*^2^ = 0.26, *p* = 0.038), confirming that less time spent on an item leads to its refixation (Figure 6C).

All these findings support an information-seeking model that parallels animal models of foraging. An explanation of some of these results could be that refixations are guided by the strength of some memory trace. Is there any specific evidence that *information* is in fact the driver of attention? To answer this, we must examine how information gain depends upon the actual numbers seen.

### Bayesian estimate of EV and risk for early fixations

While information accumulation is described by Equations (3) and (4), deciding where next to look requires a normative rule governing attention. Such a rule would specify how attention is driven by the *distributions of the estimated decision variables*, as they evolve over the decision period. We postulate that visiting and re-visiting of locations *optimizes information gain*. Similar information-guidance rules for attention have previously been proposed for low-level feature searches (Renninger et al., 2007; Hou and Zhang, 2008). In the context of choice, we expect attention to be specifically guided by uncertainty in *EV*.

**Figure 7:**
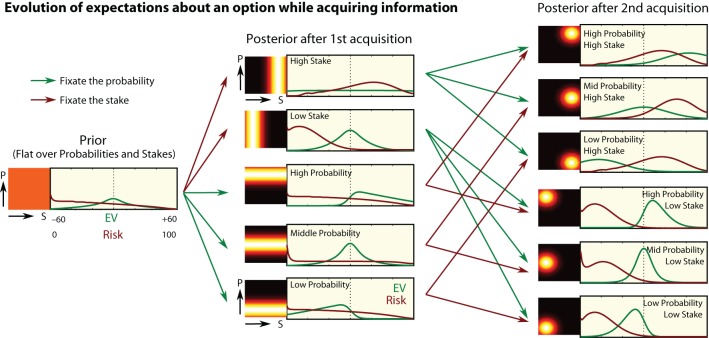
**Bayesian updating of expectations**. What information is obtained in the first two acquisitions? Heatmaps on the left of each panel illustrate the participant's estimated distribution of probability and stake. From this we calculate the estimated distribution of *EV*s and Risks Equations (6) and (7). Far left: the priors give a relatively flat distribution for *EV* and risk. First column: after the first acquisition, either a probability or stake is seen, narrowing the distribution in that dimension, and altering the density of *EV* and risk. Second column: after the first acquisition, the participant shifts attention to the other value in the same option, and his estimate of *EV* and risk improves again. As more information is accrued by fixations, the distributions become more peaked.

For the first two fixations of a gamble, we follow step-by-step the best estimate of *EV* and risk, by tracking the evolution of the Bayesian density for the *EV* and risk. We start with a flat prior, representing the lack of knowledge about the items on screen (qualitatively similar results apply if the prior is taken over all actually presented trials). After a single fixation, either the probability or the stake is known with greater precision, illustrated here as a gaussian distribution (Figure 7, heatmap to left of distribution).

If a *stake is seen first*, the density over stakes is transformed from the flat prior, to a peaked posterior (Figure 7, left and middle columns). We approximate this as

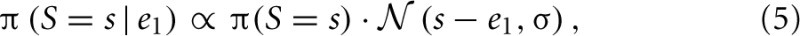



where π(*S* = *s*) = $\frac{1}{100}$ is the prior and π(*S* = *s* | *e*_1_) is the posterior over stakes after the stake value *e*_1_ is seen.

The intuition is that participants do not know for certain what number is displayed, but a narrower distribution represents having more precise knowledge. Similar belief-updating methods have recently been used for locating targets in machine vision (Butko and Movellan, 2008) and inferring word identity in reading (Bicknell and Levy, 2010).

Importantly, participants can now form estimates about the *EV* and risk:

(6) $$\begin{array}{l} \pi \left(EV=v|e_{1}\right)=\int \pi (P=p)\cdot \pi \left(S=\frac{v}{2p}|e_{1}\right)dp\\ \text{ Posterior probability}=\text{probability of }S\cdot (2P-1)\\ \text{ of }EV\text{ being }v\text{ being equal to }v; \end{array}$$

(7) $$\begin{array}{l} \;\pi \left(R=r|e_{1}\right)=\int \pi (P=p)\cdot \pi \left(S=\frac{1}{2}\sqrt{\frac{r}{p(1-p)}}|e_{1}\right)dp\;\;\;\;\;\\ \text{Posterior probability }=\text{probability of }4S^{2}P(1-P)\\ \text{ of risk being }r\text{ being equal to }r. \end{array}$$

These follow from combining Equations (1) and (2) with the posterior of (5). This captures the notion that after seeing a high or low stake, participants update their expected winnings and risks.

After a *second* fixation within the same gamble, participants acquire information about the probability *e*_2_, and the new estimated density of the probability P is given by

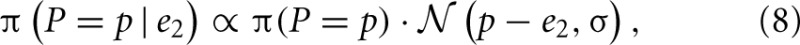



with the prior π(*P* = *p*) = $\frac{1}{100}$. Putting π(*P* = *p* | *e*_2_) in place of π(*P* = *p*) in Equations (6) and (7) gives the new posteriors for *EV* and risk after the second fixation, π(*EV* | *e*_1_, *e*_2_) and π(*R* | *e*_1_, *e*_2_) (Figure 7, right column). This posterior now incorporates the fact that participants have some knowledge about both the stake and probability to estimate what they can win.

After the first fixation on the stake, should participants fixate the probability of the same option? We quantify how much information can be gained by looking at the probability, using an information metric. The expected information gained about *EV* (the gain from a within-option saccade, i.e., vertical saccade) could be measured in bits as the average over possible values of *e*_2_ of

(9) $$\begin{array}{l} \text{ Information}=D_{KL}\left[\pi \left(EV=v|e_{1},e_{2}\right)||\pi \left(EV=v|e_{1}\right)\right]\\ \;=\int \pi \left(EV=v|e_{1}\right)\cdot \log\;\left(\frac{\pi \left(EV=v|e_{1}\right)}{\pi \left(EV=v|e_{2}\right)}\;\right)\;dv.\;\;\;\;\;\\ \text{Information gain}=\text{distance between probability distributions}\\ \text{ before and after seeing an item}. \end{array}$$

Intuitively, if gazing at a location could dramatically change the distribution of possible *EV*s, then that location is potentially very informative. That is, informativeness is defined as the distance between the current and possible future distributions of *EV*.

**Figure 8:**
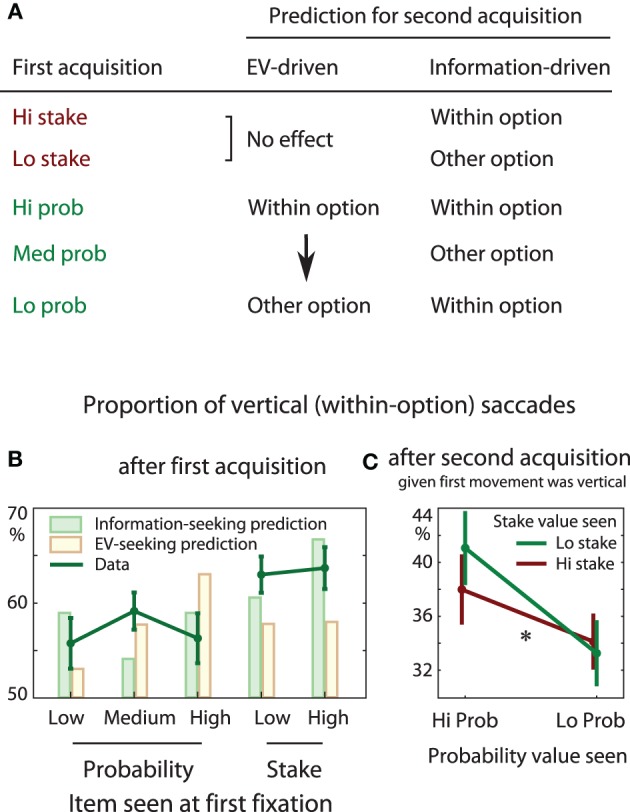
**(A)** Attention may be guided by *EV* or by information seeking. The two drives predict different patterns of fixation in our task. If attention were *EV*-seeking, gaze ought to remain within the current option if the first-seen item was a high probability, but not if it were a low probability. On the other hand, if attention were information-seeking, gaze ought to remain within the current option if a high stake was seen, compared to a low stake. **(B)** After the first fixation, participants may look vertically within the option, or across to the other option. Where they look next depends on what they just saw: within-option saccades are commoner after seeing a stake. This is predicted when attention is information-driven, rather than *EV*-driven. Green bars represent the theoretical information gain from making a within-option saccade, calculated as 〈*D_KL_* [posterior EV || prior EV]〉_prior p,s_, which represents how much information one could expect to learn after making a particular saccade. Yellow bars represent the Bayesian estimate of *EV* of the current option. Both green (information) and yellow (*EV*) bars are arbitrarily scaled. **(C)** On trials where the first two acquisitions were within one gamble, participants sometimes refixate the first item seen. This is more likely when the probability was high (*p* = 0.047), but there was no effect of stake (*p* > 0.05, with no interaction).

Analogous results are found when a probability is fixated first. The information gained by remaining within an option is shown in Figure 8, and is characterized as follows:

(P4) If the first item seen was a stake, more information is gained by remaining within the same gamble, than if a probability was seen.
(P5) More information is gained if the stake seen was high, compared to low.
(P6) If the first item seen was a probability, it is more informative to remain within the same gamble if the probability was high or low, than when it is close to 50%.

These features are robust to differing amounts of information per fixation (changes in σ). We took σ = 15 for the residual uncertainty about a number after it is fixated once. Note that predictions P4–P6 (predicting fixation sequence) are independent of P1–P3 (predicting fixation duration), because the Bayesian updating in its present form ignores fixation durations and decay. A composite model incorporating both decay and time-dependent updating could be used, which would generate all six predictions P1–P6, but would require fitting of accumulation and decay rate parameters. Instead, we chose to split the two aspects of the model to allow for more straightforward testing.

### Is the first shift of attention driven by EV or information?

After the first acquisition, attention could either be directed within the gamble to the other number (vertical saccade), or across to the opposite side gamble (horizontal or diagonal). If attention were driven by expected value, after the first fixation, we would expect participants to look within an option after seeing a high probability, but not after a low probability, and no effect of stake size (seeing a high stake indicates a high risk, but without informing about the expected value). This prediction is illustrated by the bars in Figure 8B. On the other hand, if information guides attention, we should expect high stakes to cause more within-option saccades than low stakes—because the higher the stake, the more informative is the corresponding probability.

We found that overall participants were generally more likely to look within the current gamble (60% preponderance). If the first fixation was on a stake, participants were more likely to look within the option, compared to when they first fixated a probability [63 vs. 57%, *t*_(16)_ = 2.17, *p* = 0.046, Figure 8B]. This is consistent with an information-seeking account of attention, since stakes initially provide no information about *EV*, whereas probabilities do. However, we did not find an effect of the *magnitude* of the probability or stake first fixated (*p* > 0.2). Comparing these result with optimal information-seeking (previous section) shows that, in our participants, attention seeks information according to criterion (P4), but not (P5) or (P6), for the first gaze shift.

### What drives refixations on the second shift of attention?

Next, we examined only trials where the first two acquisitions were both within one option. At this point participants had seen *both* the stake and probability of one option. Subsequent saccades could either be vertical, refixating the first value seen, or could go across to the other option.

Equations (5)–(9) describe how informative the next shift of attention would be, given the estimates at the end of the second acquisition.

If refixation were driven by *EV*, we might expect more of these refixations when a high probability and a high stake were seen, and fewer when a low probability and high stake was seen. Note that a pure information-seeking account would always predict moving to the other option. We found that on average participants immediately refixated in 37% of trials, and there were more refixations when the probability was high than when it was low [main effect of option probability, *F*_(1, 48)_ = 4.15, *p* = 0.047, Figure 8C]. This is consistent with a *pull of the higher EV*, and demonstrates that the second shift of fixation is not simply random. As expected there was no effect of the stake size (*p* = 0.7). However, we did not find the expected interaction between the probability and stake (*p* = 0.49): high stakes did not increase the drive of probability.

In these analyses of the first and second shifts of attention, we included color coding as a factor. There was no main effect of color coding, and no interaction (*p* > 0.05). Since we had only expected color coding to be relevant for the first two shifts of attention, we collapsed across color conditions for the following analysis of later fixations.

### Subsequent timecourse of attentional control by EV and information

**Figure 9:**
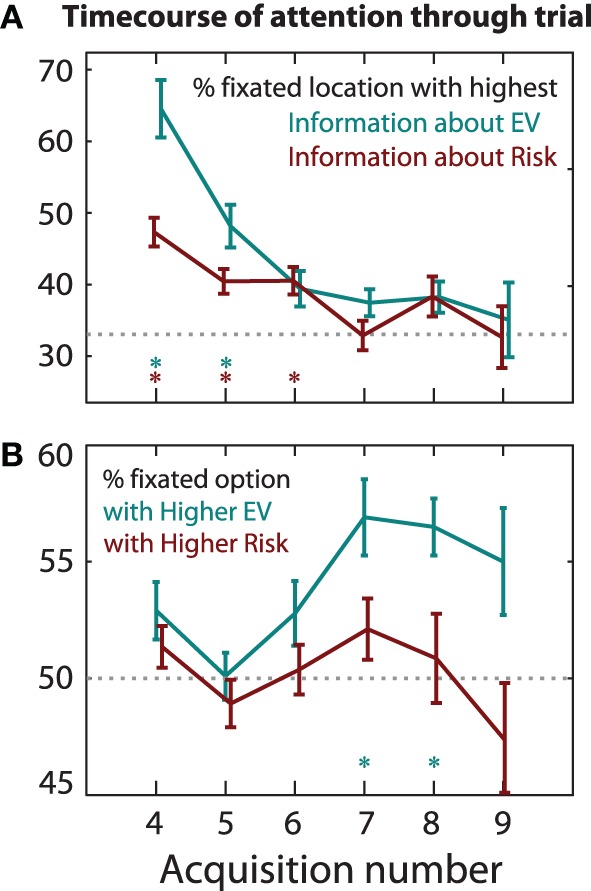
**Timecourse of attentional control. (A)** Early on in a trial, attention is drawn by information. There is a strong pull by information about expected value, as calculated by Bayesian updating. The y-axis shows how often participants' saccades coincide with the information-seeking prediction. This falls to chance (33%) after the sixth acquisition in a trial. There is a weak effect of information about risk. Asterisks denote acquisitions when gaze was significantly drawn toward the highest information, relative to chance (*p* < 0.05). **(B)** Participants increasingly tend to fixate on the option with higher *EV* through a trial. For both **(A,B)**, *EV* and risk estimates for participants' fixation sequences π(*EV* |*e*_1_, *e*_2_ … *e_i_*) were calculated using Bayesian updating rules using σ = 15.

Information seeking only partly predicts the first two shifts of attention. For subsequent fixations, however, it is more effective. We can follow the acquisition sequence that participants made, iteratively applying Bayesian updating Equations (5)–(9). At each fixation, we calculated the online estimate of the option *EV*s and risks, assuming a fixed amount of information about the number is acquired on each acquisition, with no forgetting. The expectation of information gain Equation (9) gives us the optimal next saccade to maximize information—either information about *EV* or risk. Figure 9A shows on each fixation, whether or not participants fixated the “best” item in order to maximize information about *EV*, or risk. On the fourth and fifth acquisitions in a trial, attention is strongly drawn toward the higher information location, but on later acquisitions only weakly so [compared to chance, *t*_(16)_ > 2.79, *p* < 0.05 correcting for 24 multiple comparisons].

What was the timecourse of the attentional pull of *EV*? Early acquisitions were equally likely to go to the lower or higher *EV* option, whereas later acquisitions (7th and 8th) tended toward the higher *EV* option [Figure 9B, *t*_(16)_ > 2.15, corrected *p* < 0.05, up to 58% to higher *EV*; qualitatively similar results were obtained using σ = 5, 15, or 60]. Thus, value had a stronger pull later in the decision period.

**Figure 10:**
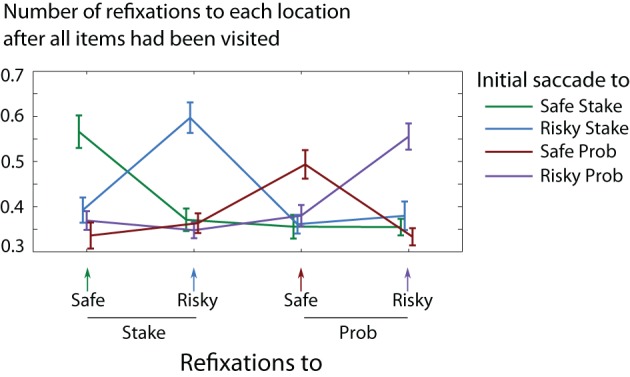
**The chance of refixating each displayed item**. Line colour indicates which item was fixated first on a trial. Refixations are strongly drawn to the very first acquisition of the trial.

The acquisition immediately after all locations had been visited was strongly drawn toward the *initially fixated* item (Figure 10), despite initial the initial fixation being at chance to each item type; this is precisely what would be expected from the decay of information.

To rule out possible bias due to there being more acquisitions in trials with lower and more similar *EV*s (see below), we aligned each trial's acquisition series to the *end* of the sequence, such that all the final, penultimate etc. acquisitions were grouped. Again, the effect of *EV* increased monotonically through the trial, to the final saccade which had a 62% chance of going to the higher *EV*. The final saccade correlated with choice on 80% of trials (Figure 5).

The results suggest that both uncertainty and *EV* can drive attentional shifts, but at different points in a trial. A possible attention-guiding rule might be to maximize some linear combination of informativeness and estimated expected value, where the weighting changes through the trial:

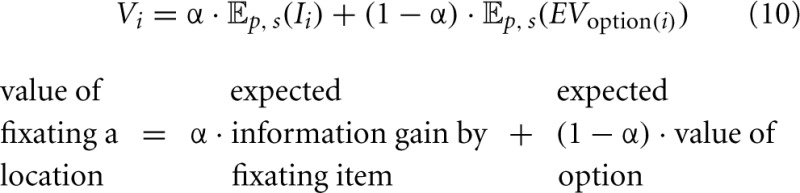



over the three possible shifts of attention. Here *V_i_* represents the intrinsic worth of a given shift of attention, *I_i_* is its information gain given by Equation (9), and *EV*_option(i)_ is the estimated reward *EV* of the corresponding option. The coefficient α(*t*) might begin at 1, and decrease to zero through a trial, weighting first information then value.

### Amount of foraging for information depends on EV and risk

**Figure 11:**
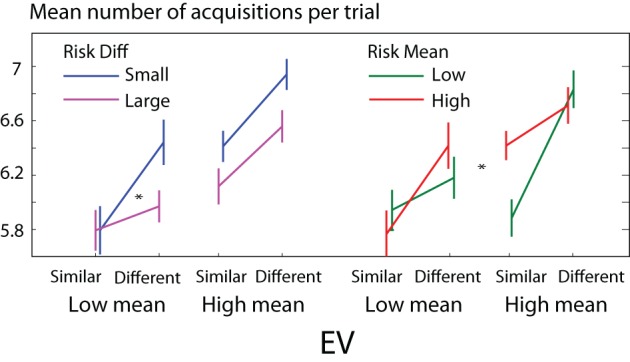
**Participants make more acquisitions on trials where the mean *EV* of the two gambles is low, and when the two *EV*s are similar (higher difficulty)**. The presence of a large risk difference reduces the difficulty effect (interaction *p* < 0.05). High mean risk increases the difficulty effect when mean *EV*s are low, but decreases it when mean *EV*s are high (interaction *p* < 0.05).

We quantified foraging for information by the number of acquisitions (changes of fixation quadrant) before choosing. Participants made more acquisitions when the expected values of the gambles were *both low*, than when they are both high [ANOVA, median split factors: mean EV, *EV* difference, mean risk, risk difference; main effect of mean *EV*, *F*_(1, 16)_ = 13.4, *p* = 0.0038]. They also made more acquisitions when the *difference in expected values* of the two gambles was *small* (Figure 11), i.e., harder decisions led to more exploration [main effect of *EV* difference, *F*_(1, 16)_ = 8.96, *p* = 0.0086]. This would be consistent with estimated distributions of value getting progressively sharper, or more accurate, with more information: sharper posterior distributions are required in order to distinguish between options with similar *EV*s, as predicted by diffusion and rise-to-threshold models (Carpenter and Williams, 1995; Ratcliff and Smith, 2004). When the two risks were similar, the number of acquisitions was strongly modulated by *EV* difference. But when the two *risks were very different*, *EV* had little effect [interaction of risk difference with *EV* difference, *F*_(1, 16)_ = 5.53, *p* = 0.023) (Figure 11).

**Figure 12:**
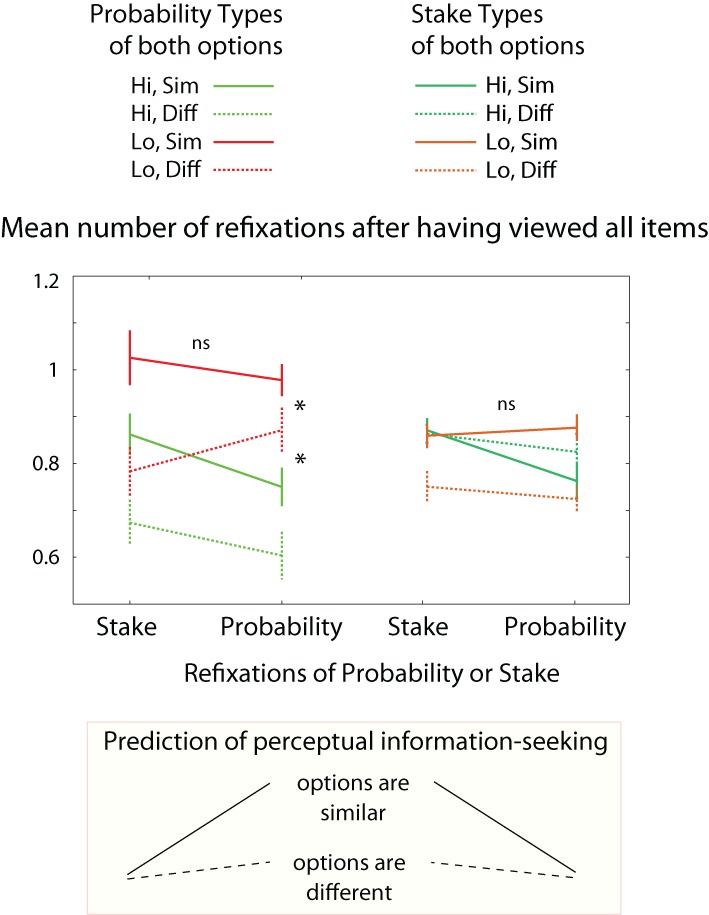
**Information about number identity or option *EV*?** When probabilities are similar, or stakes are similar, the decision is harder because more precise information is required to distinguish the options. Accordingly participants make more acquisitions by refixating. However, if the probabilities are similar, we do not find increased refixations specifically of probabilities; likewise when stakes are similar, we do not see increased refixations specifically of stakes. This suggests that the representational level that directs attention is not a perceptual or numerical level, but rather, integrated *EV* and risk of the options. Asterisks: 3-way ANOVA *p* < 0.05.

Are these similarity-driven refixations specifically targeted to the most informative locations? If refixations were attracted by information about individual display items, we would expect participants to refixate probabilities when the probabilities are similar, and stakes when the stakes are similar. However, this effect is not seen (Figure 12, left). Participants *do* make more refixations when the probability difference is small, but the extra refixations are not specifically directed to the probabilities [main effects of mean probability and probability difference, *F*_(1, 112)_ = 22 and 28, respectively, *p* < 0.001, but no interaction with which item was refixated). Similar stakes also increase refixations compared to different stakes, but again a *general* increase of refixation is seen, not specific to the stakes [effect of stake difference *F*_(1, 112)_ = 0.03, Figure 12 right]. This finding suggests that the comparison takes place not in feature-space, but in value-space: both the probabilities and stakes are counted as informative, when comparison of either is difficult.

## Discussion

We designed a task in which participants could freely acquire information before making a decision. Two options were inspected, each of which had a monetary stake and a probability of winning vs. losing that stake. Unlike standard decision-making paradigms, we examine the trajectory of attention (indexed by eye position) before the choice is made. After freely acquiring information, participants made a button press choice. We found that they frequently refixated items, even before visiting all four locations. Early in a trial, the trajectory of attention was directed to locations with the highest information gain. Later on, attention was guided to the option of higher expected value (Figure 9).

Why would locations be refixated? We interpret the findings in terms of foraging: choosing an option involves first approaching the option, then deciding whether to accept or reject that option. Early in the trial, under uncertainty, attention is directed to high-variance options, in an attempt to resolve their uncertainty by acquiring information. As information accumulates, however, attention becomes progressively guided by reward value, such that an “engage/search” strategy could be used to make the best choice.

The temporal pattern of the attentional trajectory provided support for an information foraging mechanism:

- First, dwell times were shorter later in a trial. We suggest that this is because later in a trial, information is sampled in smaller aliquots. This is predicted by a leaky accumulator (Ratcliff and Smith, 2004), in which evidence about an item's identity increases while it is fixated, but decays when it is unfixated (Figure 1); fixations are terminated when information reaches a threshold.
- Second, information foraging also predicts that refixations are shorter than first-fixations, at the same serial position—a prediction that runs in parallel to predictions of classical foraging theory (Waage, 1979).
- Third, since the total quantity of information obtained increases with acquisition duration, the model also predicts that the chance of refixating an item falls according to its initial dwell time.
- Fourth, foraging predicts that participants will generally be looking at the chosen option when they make the button press—which is true in 80% of trials—since the final choice is in fact an “accept/reject” decision.
- Finally, assuming that participants choose to look at uncertainty, the model also correctly predicts that the first item fixated is most likely to be refixated once all items have been viewed (an effect also predicted by inhibition of return).

But is the assumption of looking toward uncertainty warranted? If attention were guided solely by information seeking, we would not observe biases of looking toward reward (Ding and Hikosaka, 2007; Milstein and Dorris, 2007; Hickey et al., 2010). On the other hand, If attention were guided solely by reward, we would not learn about our environment (Hogarth et al., 2008).

### Two competing hypotheses for goal-driven guidance of attention: sharpening perception vs. sharpening value representation

According to a *perceptual model*, attention should favor objects whose identity is uncertain. This is the prediction of models in which attention aims to improve the precision of our internal representation of causes in the world, e.g., a free energy formulation of perception. A competing model is that attention favors objects which inform us about *expected value* (Milstein and Dorris, 2007). For example, if an object is likely to indicate what the value of an option is, it should command attention. Here, attention aims to improve informed choice, and attentional trajectories are computed in terms of option-value precision, as opposed to perceptual precision. Perceptual information-seeking is agnostic of the actual numbers seen. On the other hand, *EV*-based information-seeking predicts that revisiting patterns should depend on the actual numerical values. Such effects are seen in our data (Figures 8B,C and 12), consistent with the possibility that the initial trajectory of attention is computed to reduce uncertainty in *option-value* space, rather than perceptual space, using an information-maximizing principle. This could in principle be implemented using an active inference framework. This distinction provides a new way to disentangle different levels of “top-down” attentional control: in our task, the eyes are directed not simply to perceptual uncertainty, but to option value uncertainty.

Our results thus lead us to consider that *value* uncertainty is more likely to be relevant than *perceptual* uncertainty, in this task. Numerical values may be subject to similar noisy integration to qualitative stimuli (Krajbich et al., 2012) Such a proposal would be consistent with evidence that numerical magnitude representations in the parietal lobe are limited in their precision, in contrast to precise symbolic representations present during immediate perception (Naccache and Dehaene, 2001; Brannon, 2006).

### Explaining refixations

Refixations, we argue, occur because of incomplete knowledge of previously visited items. This could be due to poor retention or poor acquisition. Although retention is generally considered to have a capacity of 4 or more items (Snyder and Kingstone, 2000; Gilchrist et al., 2001), a variable-precision account of working memory retention might predict refixations, particularly when combined with temporal decay (Bays and Husain, 2008; Zokaei et al., 2011). A more straightforward explanation of refixation is that participants only *acquire* a limited amount of information about each target as they fixate it. This can be expressed as incremental changes in the estimated probability density over the four display values (Figure 7). The gain of information may depend on fixation duration, and subsequently information may decay (Figure 1).

To explain refixation patterns, we invoke a concept of “*infomation salience.”* The information content of a stimulus can be quantified as the distance between probability densities over *EV* before and after an item is identified. Thus, information content indicates the reduction in uncertainty that a stimulus might bring when identified. The concept of information salience is meant to describe the way in which attention can be captured by this informativeness, even when other accounts (inhibition of return, Posner and Cohen, 1984; Itti and Koch, 2001) predict it should not. Our task allows us to quantify mathematically what has been called “attention to the unknown” (Gottlieb, 2012), and compare it directly with other attentional biases, including perceptual salience and reward.

One old candidate for explaining attention to the unknown, is *inhibition of return* (Rafal et al., 1989). IOR has long been thought of as an aid to foraging in an environment (Klein and MacInnes, 1999; Gilchrist and Harvey, 2000; Klein, 2000; Hooge et al., 2005), and has inspired dynamic models of sequential attentional selection (Itti and Koch, 2001; Hou and Zhang, 2008). IOR both slows and prevents returning saccades (Bays and Husain, 2012), and in this way, may function as a novelty bias.

Of interest, one study has shown IOR to be contingent upon the occurrence of reward and dependent upon medial frontal cortex (Hodgson et al., 2002). IOR may persist for up to 5 previously attended locations (Snyder and Kingstone, 2000); its duration is increased by amphetamine and may be reduced in Parkinson's disease (Filoteo et al., 1997; Poliakoff et al., 2003). It also varies between individuals according to DAT1 gene polymorphisms (Colzato et al., 2010). Frontal dopaminergic mechanisms are thus likely to be crucial in generating the drive of spatial attention toward reward value or uncertainty.

Although IOR explains why refixations go toward locations that haven't been recently fixated, it makes no predictions about (1) the relationships with fixation durations, (2) the first couple of acquisitions, nor (3) the effect of the actual numeric values seen. However, specific predictions are made by information foraging coupled with Bayesian updating of *EV*s.

### “Deciding” where to look

Many authors have considered saccadic control as a surrogate for decision making (Glimcher, 2001; Gold and Shadlen, 2007). From our results we argue, in contrast, that deciding where to attend involves *different* considerations to deciding upon actions:

- Attention, unlike action, is also guided by bottom up-salience (Theeuwes, 2010), does not result directly in primary reward (Maunsell, 2004), does not carry a sense of agency, and has a different kind of cost than the effort required for actions (Haith et al., 2012).
- These functional differences may be manifest neurally. Values for action and values of stimuli appear to be represented in distinct prefrontal regions (Rangel and Hare, 2010). Orbitofrontal representations of *stimulus* value are modulated by attention (Lim et al., 2011) and by choice selection (Padoa-Schioppa and Assad, 2006). On the other hand, dorsomedial representations of *action* value are modulated by conflict, error monitoring, and foraging (engage/search) strategies.

Computationally, a critical difference is that “deciding” compares values, whereas “attending” compares uncertainties. Information foraging thus requires different mechanisms to classical decision-making models of winner-takes-all competition between the option values (Wang, 2002; Wong et al., 2007). So long as more information is available in the environment, then for guiding attention, the *least certain* option needs to win out (Renninger et al., 2007). One implementation of this would be a neural map of uncertainty, rather than value, that guides attention—analogous to maps proposed for reward (Peck et al., 2009) and salience (Koch and Ullman, 1985).

Even when attention is guided by values, we suggest that the values are integrated in a fundamentally different way. Rather than comparing option values in an accumulator (Ratcliff and McKoon, 2007), we suggest that attention is guided by value via a spatial map, which may incorporate reward expectation and history from many sources (Platt and Glimcher, 1999; Ding and Hikosaka, 2007; Milstein and Dorris, 2007), such as online value estimates. Such attentional value biases are entirely compatible with action-choice being subserved by independent comparators often used in decision models.

### Concerns and limitations

Although the framework advanced here has some attractions, there are also some potential concerns or limitations. First, does *EV* really carry less weight early in a trial (Figure 9)? At the start of a trial, participants have no information about *EV*, so it is not surprising that early fixations are not directed toward the higher *EV* option. If this is the case, perhaps the relative influence of *EV* and information do not vary through a trial, i.e., the coefficient α(*t*) in Equation (10) might in fact be constant. To address this, we used the estimated posterior for *EV* Equation (4) to re-analyse whether participants fixated the option that had the higher value *according to their online estimates*, and obtained results similar to Figure 9B. Participants looked at the higher *EV* estimates on fixations 6 and 7 (corrected *p* < 0.05), but not on earlier fixations. Thus, we conclude that attention was significantly pulled by *EV* later but not earlier in the trial. We cannot rule out, however, that earlier in the trial *EV* contributes less because the estimated *EV* differences are smaller, or that later in the trial high *EV*s are fixated as a by-product of a comparison process.

Second, the first few shifts of attention (indexed by gaze) did not show true information-guidance. The second acquisition tended to be within the same gamble as the first fixation, which contravenes predictions of pure information-seeking: information gain is maximized by looking across to the other gamble. Even more surprisingly, participants refixated recently seen items before all items have been explored. For example, sometimes both the second and third acquisitions are “within-option” movements (Figure 2D, “WWA”). Contrary to this, pure information-seeking mandates that attention go preferentially to previously unseen items. Refixations ought *not* to occur until after all items have been visited, even accounting for memory limitation or “decay of information.” The unconstrained decision time in our task might have favored this suboptimal behavior in the first few saccades. In contrast, an information-seeking policy does explain later fixations (Figure 9A).

We suggest that more elaborate models of information acquisition may be needed to explain these findings. We suggest three possible extensions. First, the information-accrual rate [parameter *k*_1_ in Equation (1)] may not be constant through the decision period; in particular, it might be low for the initial acquisitions, which would also explain the longer initial fixations (Figure 4B). A second more intriguing possibility is that it is easier to integrate the probability and stake of an option when they are seen consecutively—perhaps reflecting a cost for shifting the focus of attention to items within working memory (Oberauer, 2002) or a cost for switching object files (Treisman et al., 1983). This cost could appear as an additional term in the shifting rule Equation (10). Our present data is not sufficient to distinguish these possibilities, but we note that “WW” patterns were commonest when fixating a high probability first—indicating that order of acquisition influences ease of integration. A third possibility is that saccades are not chosen to maximize information at the next movement, but rather, a whole sequence of subsequent saccades is chosen, to maximize information gain over several fixations. If we were to include decay into the updating model, fixation order would make a difference to information, possibly resulting in a different optimal strategy. Our current model assumes some form of bounded rationality, since we ignore the possibility of planning sequences.

Third, how much of attentional control can be explained by *EV* and information? The results showed that attention was significantly attracted to information salience early in a trial, and to high *EV* later in a trial. However, our maximal prediction accuracy was only 62% for information-seeking, and 61% for *EV*-seeking (Figure 9). Could other factors guide attention in our task? Of note, participants did not always choose the higher *EV*, and the final acquisition went to the chosen option on 80% of trials (Figure 5). It is likely that subjective preferences involve a more complex notion of utility than simple *EV*, for example incorporating risk preference or probability discounting (Kahneman and Tversky, 1979). These extra factors probably also contribute to attentional guidance *before* a choice.

In calculating whether participants fixated the most informative location, we took σ as constant. That is, we did not include the effect of fixation durations or decay, which would involve making assumptions about the information acquisition rate and forgetting rate. In particular, we did not fit any parameters to individual participants' performance. Information acquisition rate and forgetting rate may well vary from person to person (Colzato et al., 2010). On top of these factors, attentional guidance might itself be noisy. For example, a softmax rule (Luce, 1977) could be used to determine the next fixation location given the *EV*s and information gains. The observed transition from information salience to reward salience bears similarities with longer term switches between *exploration and exploitation* seen under risk (Daw et al., 2006; Cohen et al., 2007). In cases where information increases due to learning, the proportion of “noisy” choices that are *not* guided by value (i.e., the temperature) would decrease over time (Sutton, 1991; Carmel and Markovitch, 1999). In our case, rather than switching from random to model-driven choice, attention switches from uncertainty-seeking to reward-seeking.

Finally, throughout our analysis, we have made two assumptions: saccades are a relatively direct index of how attention is directed, and attention is focused rather than divided. Attention dissociates from eye movements in experimental conditions of enforced fixation (Juan et al., 2004), however, saccades probably entail movements of attention under most conditions (Sheliga et al., 1994; Corbetta, 1998; McPeek et al., 1999). In our displays, participants would be unable to perceive numerals that are not within a couple of degrees of fixation, as we established in pilot experiments. This enforced a serial strategy, in which dividing attention could not have been beneficial. We expect that refixations would be greatly reduced if this serial constraint were lifted, because dividing attention could facilitate both integration across dimensions and comparison within a dimension.

### Decision biases due to attention

Attention influences the decision process in a number of ways. Selecting stimulus features boosts their contribution in the stochastic progression of an ongoing decision process (Roe et al., 2001; Usher and McClelland, 2001; Kim et al., 2012). Attention may highlight supporting evidence for the favored option, generating attentional shifts within an option rather than between options (Glöckner and Herbold, 2011), but also reflecting whether a decision involves component-wise comparison or integration of value (Arieli et al., 2011). Counterproductively, attention biases choices in favor of the attended option (Krajbich et al., 2010; Brandstätter, 2011), and its influence on choice can be modeled as leaky integration of value over time, with a bias toward the attended item. These approaches show that attention powerfully modulates choice, but fail to explain how attention is itself guided.

Sampling theories make predictions about how we acquire information from the options available before a choice (Stewart et al., 2006). According to decision field theory, attention under risk is drawn in proportion to probabilities (Roe et al., 2001). But such a scenario would make attention highly inefficient at obtaining information. Optimal information gathering should not simply attend to the higher probability or expected value; rather, attention should seek uncertain options whose distribution of value has a high entropy.

It seems counterintuitive, however, to choose an option that is not being attended. Indeed participants generally choose the option they were last attending to unless that option is much worse than the other one (Shimojo et al., 2003; Krajbich et al., 2010)—but why should this be? A parsimonious explanation of this phenomenon is to regard attention as a form of foraging. Rather than deciding which item is better, decisions are made by an “engage or search” strategy. During the course of a single decision, attentional allocation dynamically switches from information-seeking to value-seeking (Figure 9). This accounts for the correlation of final saccades with both *EV* and choice (Figure 5). The decision to engage accept or reject the currently attended option might be subserved by a drift-diffusion model similar to that of Krajbich et al. (2012), which is driven by the difference between attended and unattended items.

But can we also explain the bias for choosing the initially-fixated option (Figure 5)? Information foraging predicts that after visiting all four locations, participants should refixate the first item they saw. At the same time, choice-by-foraging suggests that we choose whether or not to go for the currently fixated item, at each acquisition. Therefore, if participants begin to choose too soon—i.e., by engaging, rather than searching—we might expect the first item seen to be selected. According to this view, the first-viewed bias might be explained by premature engagement with the currently viewed option, perhaps linking reflection impulsivity to motor impulsivity (Evenden, 1999).

### Predictions of the information foraging account

The foraging view of decisions suggests that as information is “depleted from the environment”—or rather, the precision of our internal estimates approaches that of the environment—information salience no longer drives attention. At this point attention becomes driven by the estimated values. This makes two strong neurophysiological predictions. First, reward signals should propagate from stimulus-value regions early in a trial, to attentional regions later in the trial. Thus, one prediction might be that value-sensitive brain regions, such as OFC (Padoa-Schioppa and Assad, 2006; Kennerley and Wallis, 2009) encode the decision variables for each option as information is accrued, but once information acquisition begins to saturate (Figure 1) value signals propagate to parietal and oculomotor regions, biasing attention (Bisley and Goldberg, 2010). This permits a decision to accept or reject the currently fixated option, perhaps involving dorsomedial prefrontal cortex (Hayden et al., 2011; Kolling et al., 2012).

Second, in order to support information foraging, the most uncertain items in a display must compete for attention. Neural signals proportional to the lack of information or uncertainty should compete spatially, weighted by expectations of what information is available in the environment. Importantly, such competition would require not simply representation of a probability density, but rather an *explicit* representation of the uncertainty signal (Fiorillo et al., 2003; Knill and Pouget, 2004). Although uncertainty signals have been found in medial prefrontal regions (Grinband et al., 2006), as well as OFC (Hsu et al., 2005; Tobler et al., 2007; Kepecs et al., 2008; Schultz et al., 2008), the cellular representation of uncertainty remains unclear. We expect that during a decision, competition between such signals guides attentional selection.

## Conclusion

We used a freely-viewed choice between two gambles to examine the effects of risk and *EV* on the guidance of attention. We found that attention was initially drawn to uncertainty, and specifically depended on how the numbers seen determined uncertainty about *EV*. Toward the end of the trial, attention was drawn toward the higher *EV*, and eventually predicted choice. This suggests that attention is drawn by information-salience early in trials, and by reward-salience later in trials. We hypothesize that this reflects that choices are in fact made by a foraging mechanism of successively rejecting or accepting the currently attended option—a process which converges on the highest valued option.

### Conflict of interest statement

The authors declare that the research was conducted in the absence of any commercial or financial relationships that could be construed as a potential conflict of interest.
